# Supplementary material for: Purity of lithium metal electrode and its impact on lithium stripping in solid-state batteries
Source: Nat Commun. 2025 Jun 25;16:5395. doi: 10.1038/s41467-025-61006-7 (PMC12198372; doi:10.1038/s41467-025-61006-7)
Supplement: Supplementary file 1 — Supplementary Information [file 41467_2025_61006_MOESM1_ESM.pdf]

Supplementary Information

Purity of Lithium Metal Electrode and its Impact on Lithium Stripping in Solid-State Batteries

Juri Becker[1], Timo Weintraut[1,2], Sebastian L. Benz[1], Till Fuchs[1], Christian Lerch[1], Pascal Becker[1], Janis K. Eckhardt[1], Anja Henß[1,2], Felix H. Richter[1] and Jürgen Janek[1]*

[1]Institute of Physical Chemistry and Center for Materials Research (ZfM), Justus-Liebig-University Giessen, Heinrich-Buff-Ring 17, D-35392 Giessen, Germany.

[2]Institute of Experimental Physics I (IPI), Justus-Liebig-University Giessen, Heinrich-Buff-Ring 16, D-35392 Giessen, Germany.

E-Mail: juri.becker@pc.jlug.de; juergen.janek@pc.jlug.de (*corresponding)

Table S1: Summary of impurities in the two different commercially available lithium samples Li_{LP} and Li_{HP}. Contents of impurity elements are given in ppm and are based on the manufacturer's specifications.

Impurity	Content in Li_{LP} / ppm	Content in Li_{HP} / ppm
Al	5	5
Ca	400	300
Fe	50	55
K	80	80
Na	7000	820
Si	10	14

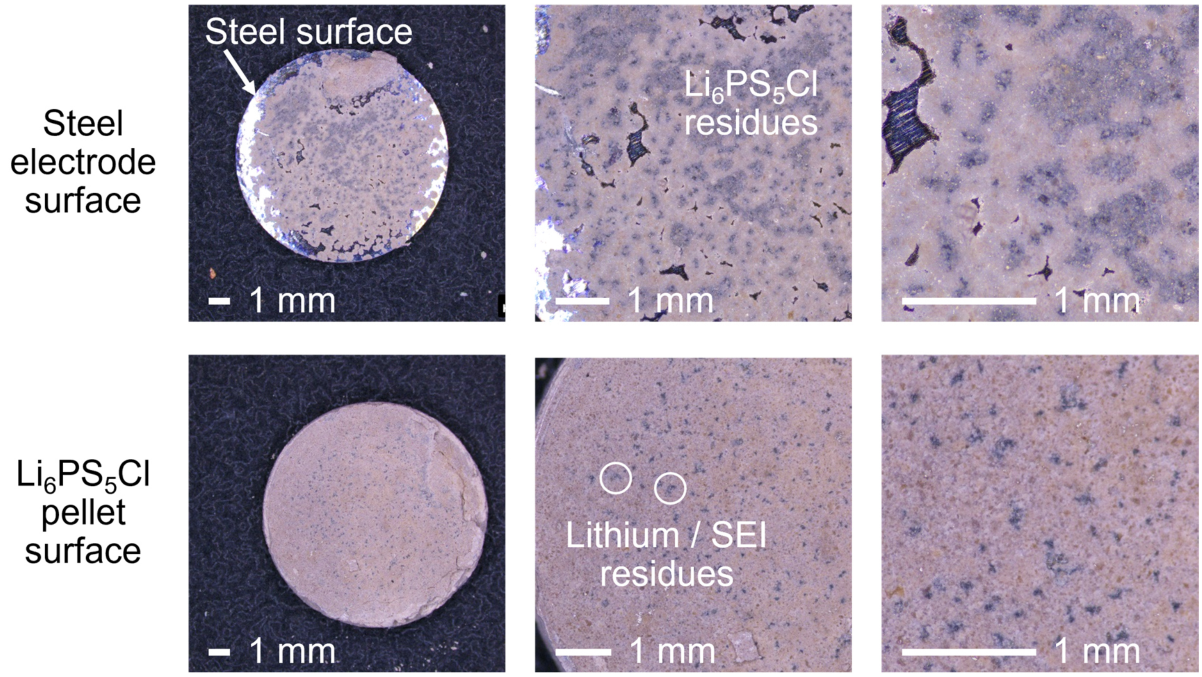

Figure S1. **Optical images of a steel working electrode and a Li_6PS_5Cl pellet surface after electrodeposition.** Please note that this sample was used for reproduction of experiments and is not the same one that was analyzed using XPS and SIMS within the manuscript. In parts where no lithium deposition has taken place, the shiny steel electrode can still be seen. However, most of the electrode is covered with residuals of Li_6PS_5Cl which adheres to the electrodeposited lithium. On the pellet surface some grey residuals of lithium or SEI products can be seen. Chipped edges on the pellet were produced during cell disassembly. This sample was analyzed ex-situ after electrodeposition of 1 mAh cm^{-2} lithium at a current density of 50 μA cm^{-2} in a reservoir-free cell system on a steel electrode.

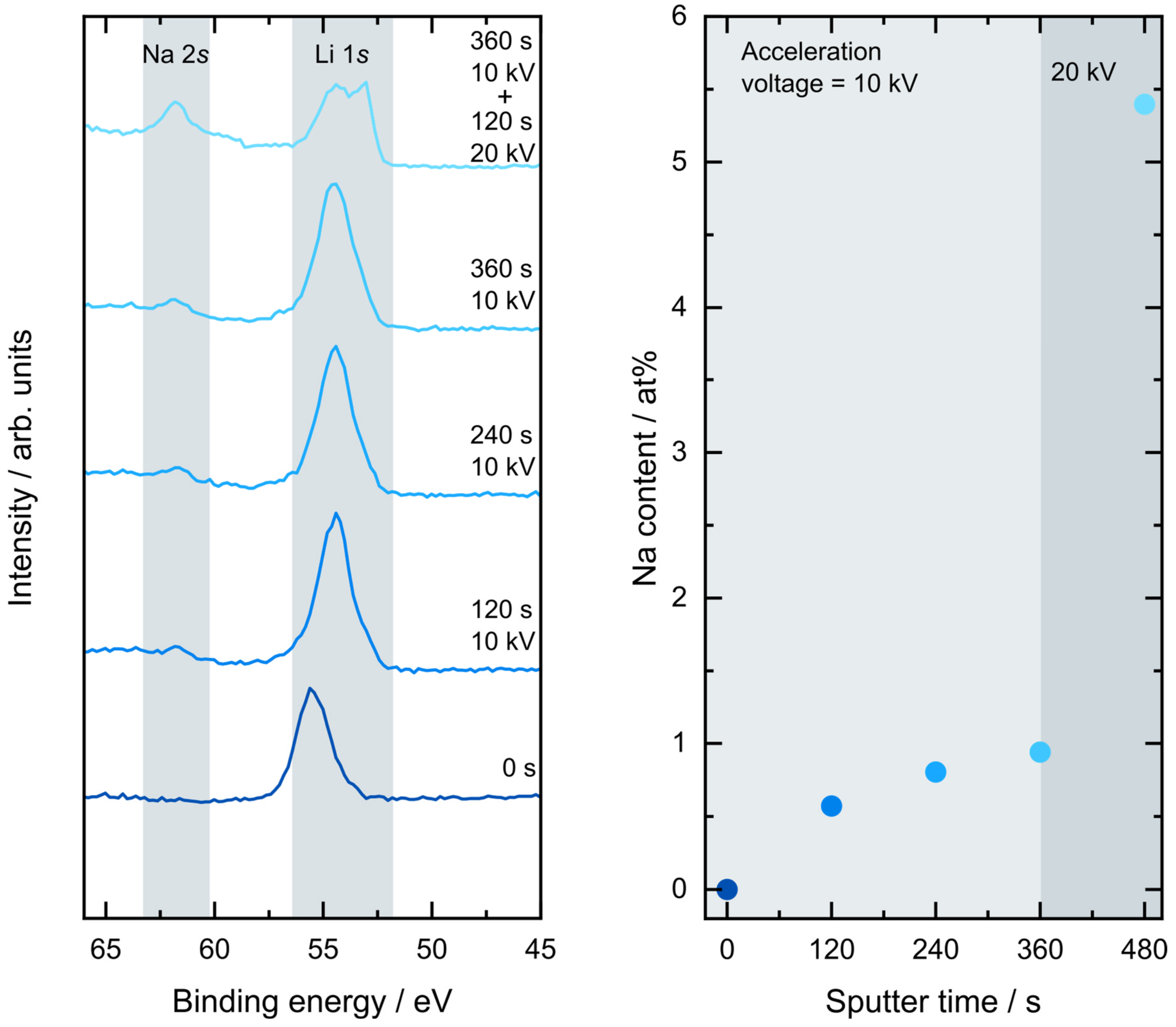

Figure S2: **The influence of argon cluster sputtering on the acquired XPS spectra.** The image on the left displays the acquired XPS spectra after the given sputtering times and acceleration voltages. Three sputter steps with an acceleration voltage of the argon clusters of 10 kV were conducted. After that, one additional sputtering step with an acceleration voltage of 20 kV was conducted. The image on the right displays the calculated sodium content (in at%) in dependence of the sputtering time.

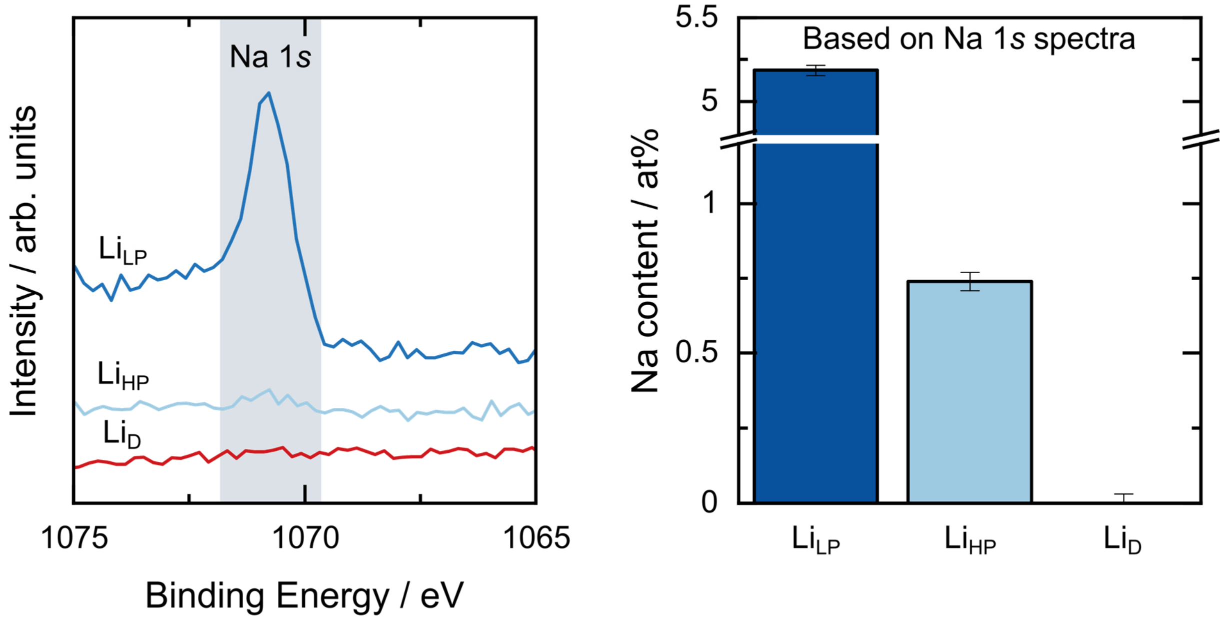

Figure S3: **XPS analysis of different samples based on the Na 1s signal.** Acquired Na 1s XPS spectra for the different lithium samples Li_{LP}, Li_{HP} and Li_D on the left. The corresponding sodium concentrations in at%, calculated from the Na 1s spectra on the left, are depicted on the right. The given error bars are based on the work of A. Shard[2], and indicate an error of 0.03 at% in all cases. In the case of Li_D no significant Na 1s signal is detectable. Therefore, only the error bars can be seen. Li_D was analyzed ex-situ after electrodeposition of 12 mAh cm^{-2} lithium at a current density of 50 µA cm^{-2} in a reservoir-free cell system on a steel electrode (see Figure 1)

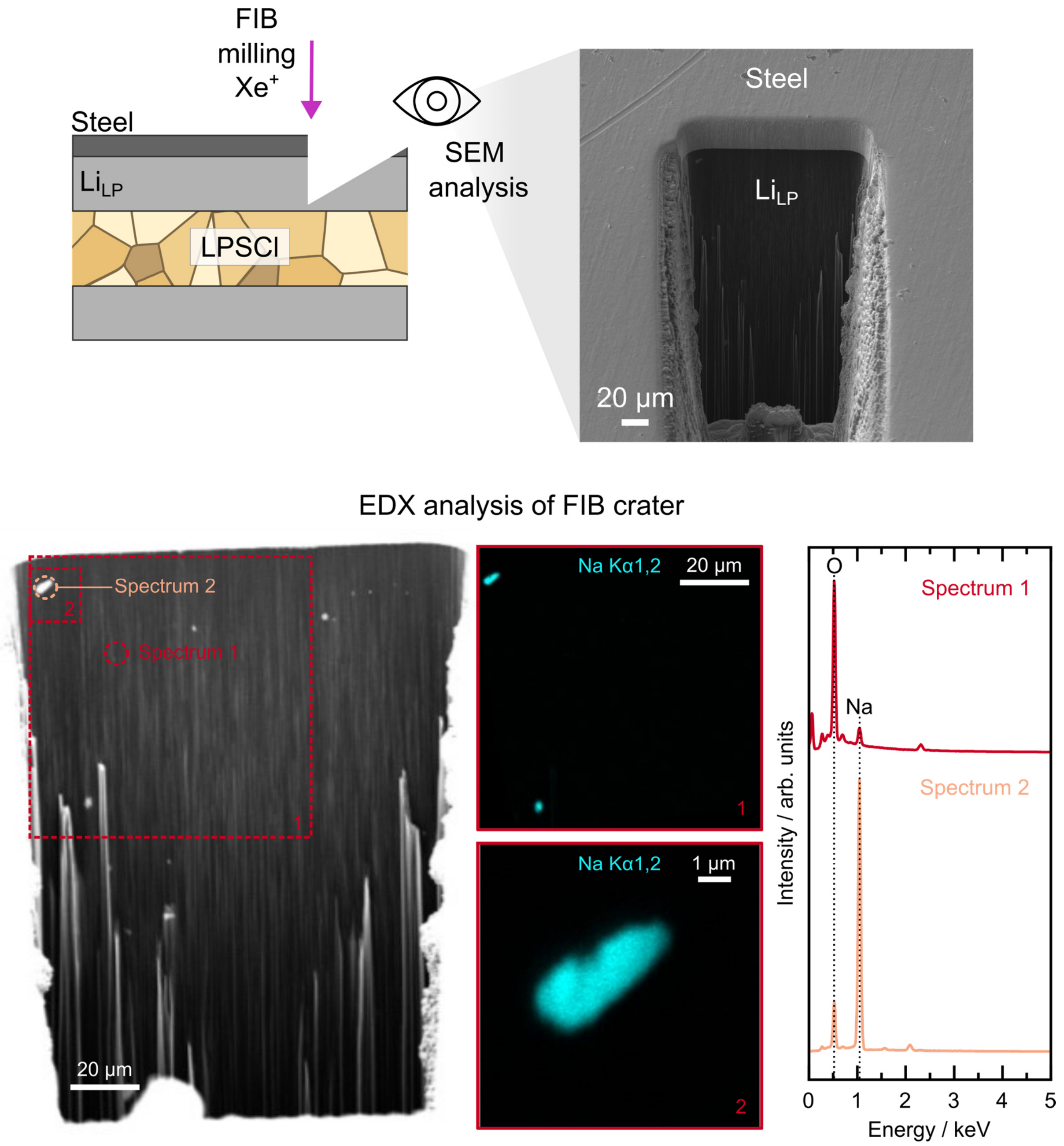

Figure S4: **Sodium precipitates within a Li$_{LP}$ electrode.** EDX analysis of a crater inside a Li$_{LP}$ metal electrode, being attached to a LPSCl pellet. As visible from the acquired EDX maps marked with red rectangles within the electron image, sodium is found in the form of a few µm sized regions inside the lithium foil. Spectrum 2 on the right side shows that the brighter appearing particles inside the lithium foil are in fact sodium precipitates. However, as visible from spectrum 1, a sodium signal is also found in areas of the sample where no such precipitates are directly visible. The sample was analyzed ex-situ after stripping of 5 mAh cm^{-2} Li$_{LP}$ at a current density of 100 µA cm^{-2} in a symmetrical Li$_{LP}$|LPSCl|Li$_{LP}$ system.

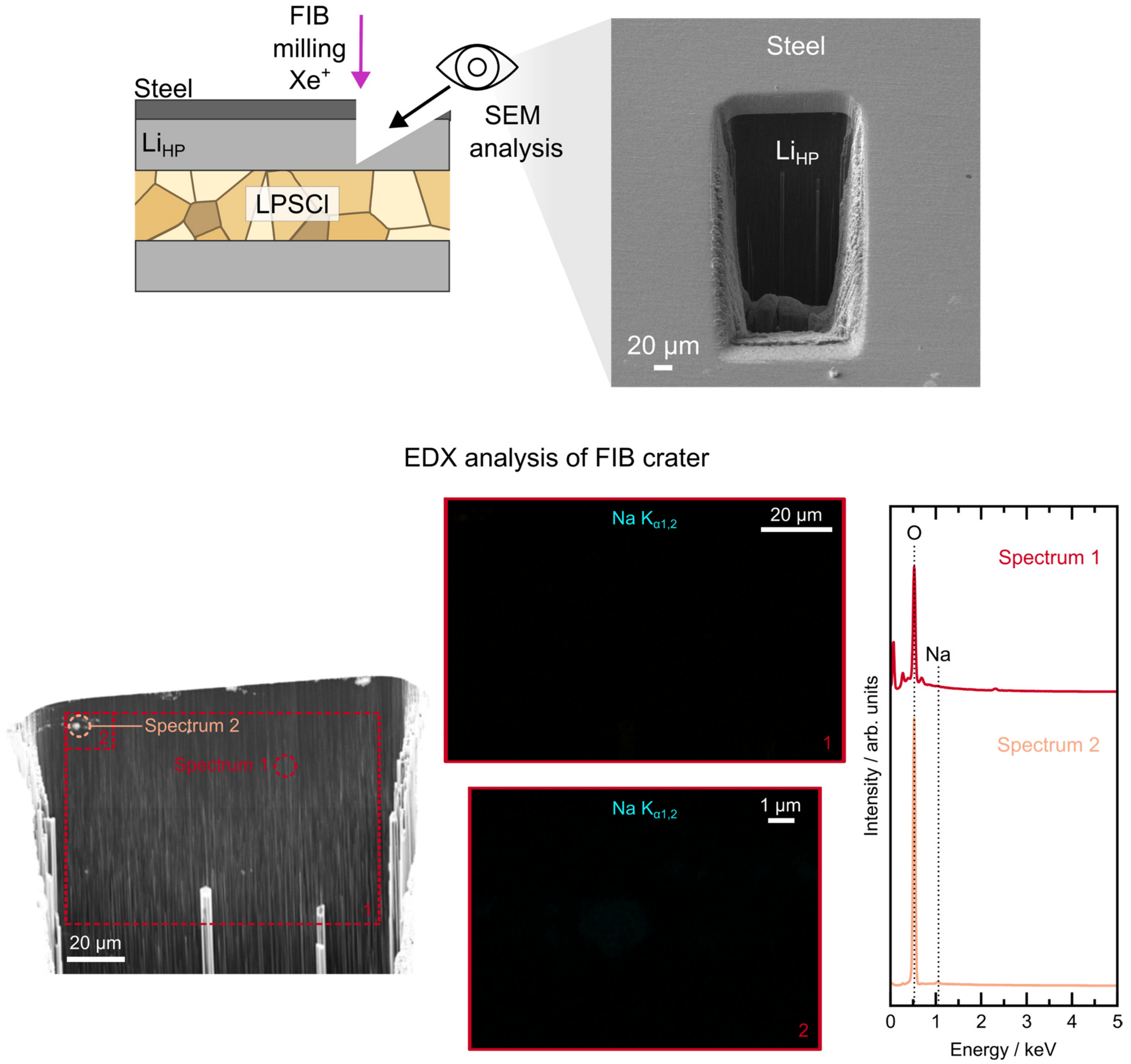

Figure S5: EDX analysis of a Li_{HP} electrode. EDX analysis of a crater inside a Li_{HP} electrode, being attached to a LPSCl pellet. As visible from the acquired EDX maps marked with red rectangles within the electron image, no sodium is found inside the lithium foil. This is further supported by two point spectra acquired in different locations inside the lithium foil. In both cases no sodium signal is found. As visible from spectrum 2, the white appearing particles within the lithium foil show a very intense oxygen signal, possibly due to some degradation products or artefacts from ion milling. The sample was analyzed ex-situ after stripping of 5 mAh cm^{-2} Li_{HP} at a current density of 100 µA cm^{-2} in a symmetrical Li_{HP}|LPSCl|Li_{HP} system.

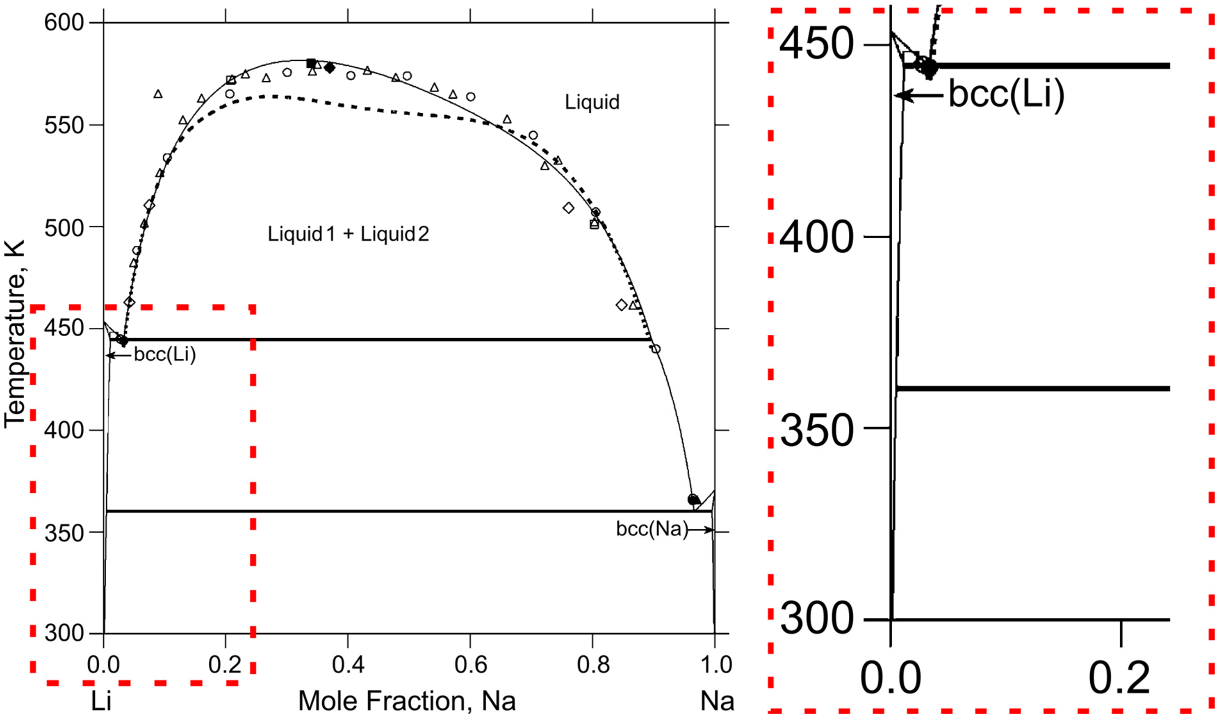

Figure S6*:* **Phase diagram of the binary Li-Na system.** The binary phase diagram of the Li-Na system is shown on the left. The magnified section on the right highlights the low sodium regime, revealing a monotectic at a sodium content of 3.4 at%. Reproduced and modified from Zhang et al.[3] Copyright 2003 Elsevier.

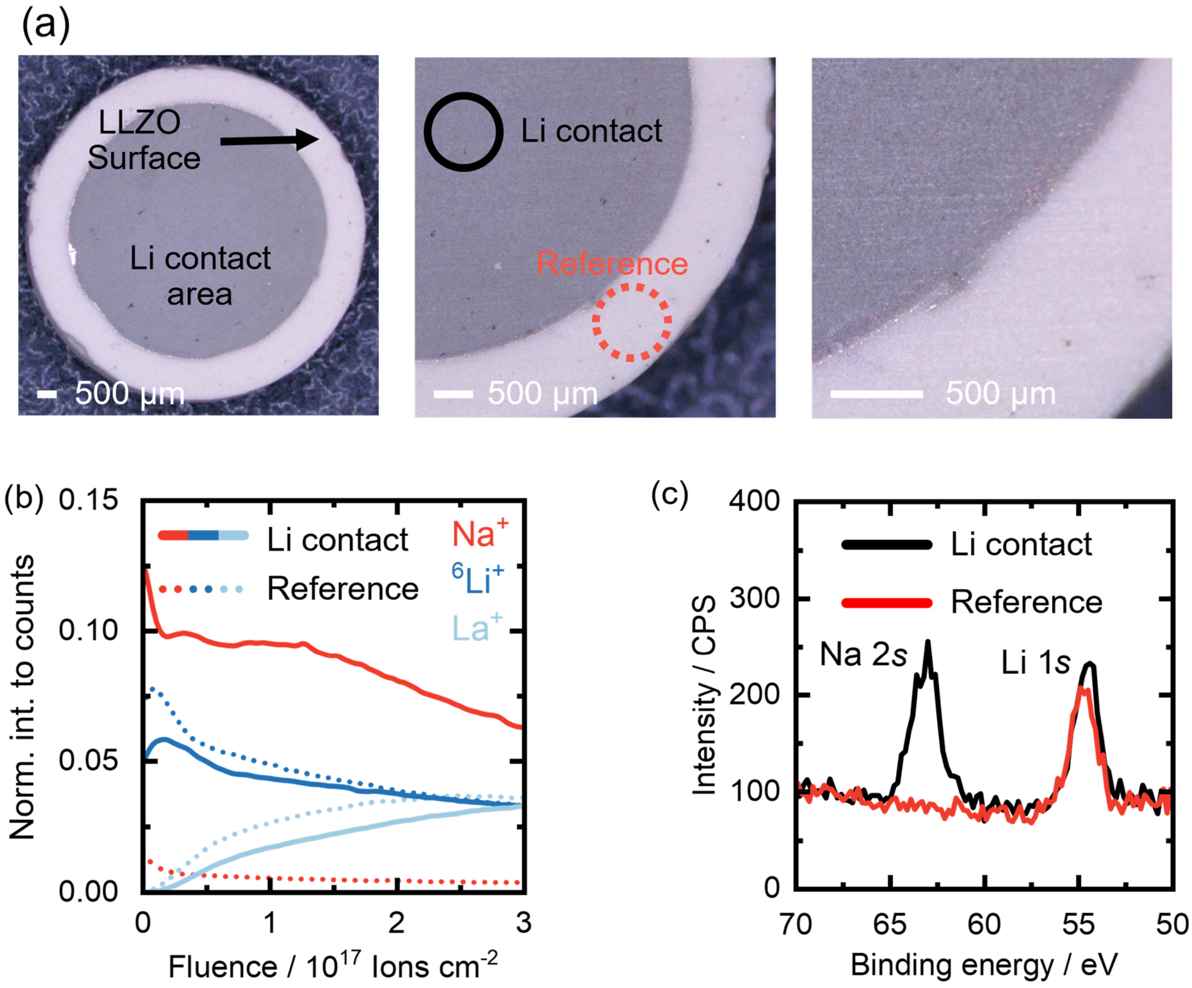

Figure S7. **Residue analysis on the LLZO surface after Li_{LP} stripping using XPS and ToF-SIMS.** (a) Optical images of a LLZO surface after lithium stripping using a Li_{LP}|LLZO|Li_{LP} cell. The lithium electrode was removed after pore formation (*i.e.*, contact loss) occurred, grey residues remain where the lithium electrode was. (b) SIMS depth profile conducted on the gray residues (straight line) and the pristine beige LLZO surface (dotted line). (c) XPS measurements on the two aforementioned areas. CPS: counts per second. The sample was analyzed ex-situ after stripping Li_{HP} at a current density of 100 µA cm^{-2} in a symmetrical Li_{LP}|LLZO|Li_{LP} cell.

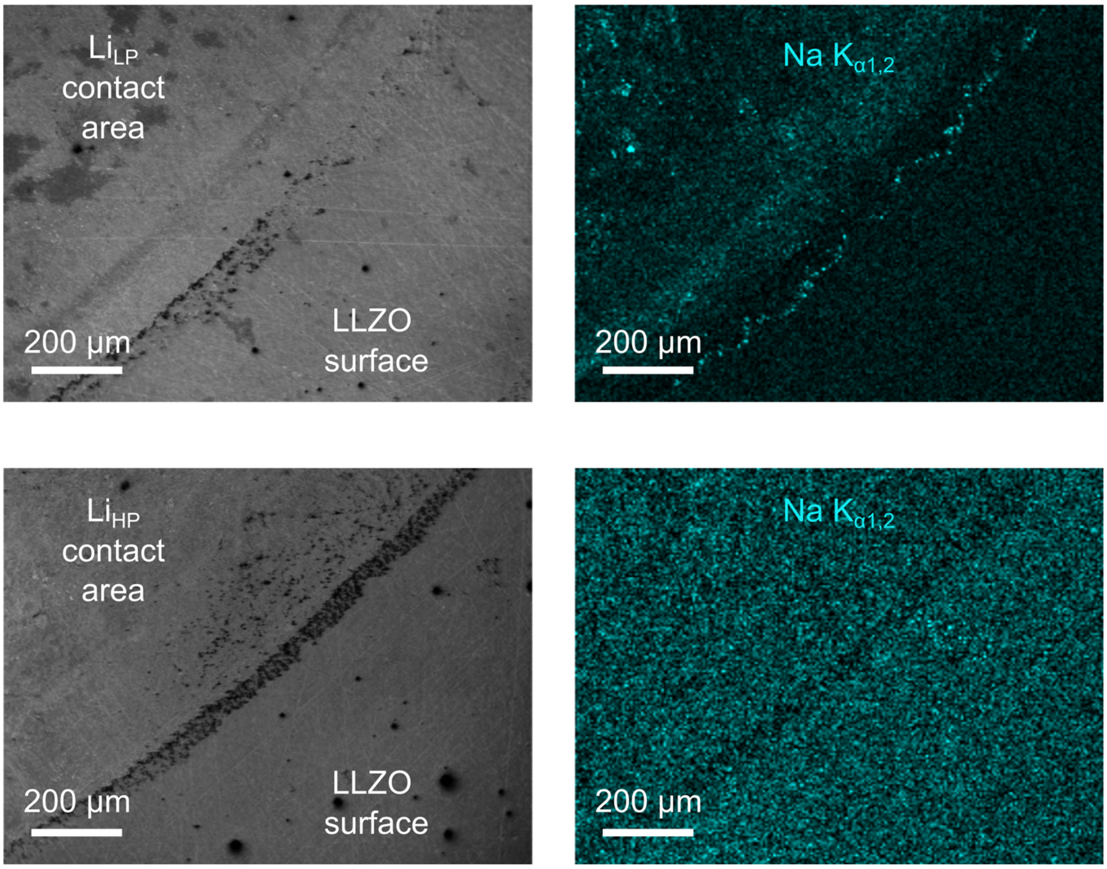

Figure S8: Residue analysis on the LLZO surface after Li_{LP} stripping using EDX. Top-view SEM and EDX analysis of a LLZO surface after being in contact with either Li_{LP} (top images) / Li_{HP} (bottom images) during lithium stripping (see Figure 4). The area, where the lithium electrode was in contact with the LLZO pellet can be seen in the left SEM images. The EDX map of the Na $K_{\alpha1,2}$ signal on the right shows an increased intensity in the area where the Li_{LP} electrode was in contact with the LLZO pellet. Conversely, for the Li_{HP} electrode a homogeneous signal is visible, being only due to noise. Please note that the intensities of the two Na $K_{\alpha1,2}$ signals have not been normalized with each other and therefore the intensities between the two images are not comparable. The sample was analyzed ex-situ after stripping Li_{LP} or Li_{HP} at a current density of 100 µA cm^{-2} for different capacities in symmetrical Li|LLZO|Li systems (see Figure 4).

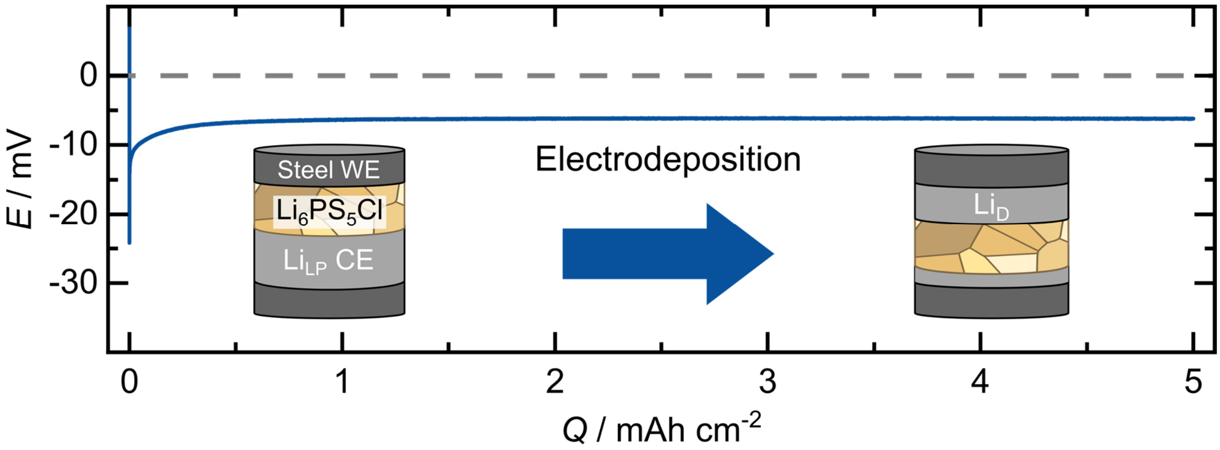

Figure S9: Electrodeposition of LiD for stripping experiments. Voltage profile during electrodeposition of Li_D for the subsequent stripping experiment (see also Figure 4). Electrodeposition is conducted with a current density of 100 µA cm^{-2} at an external pressure of 15 MPa.

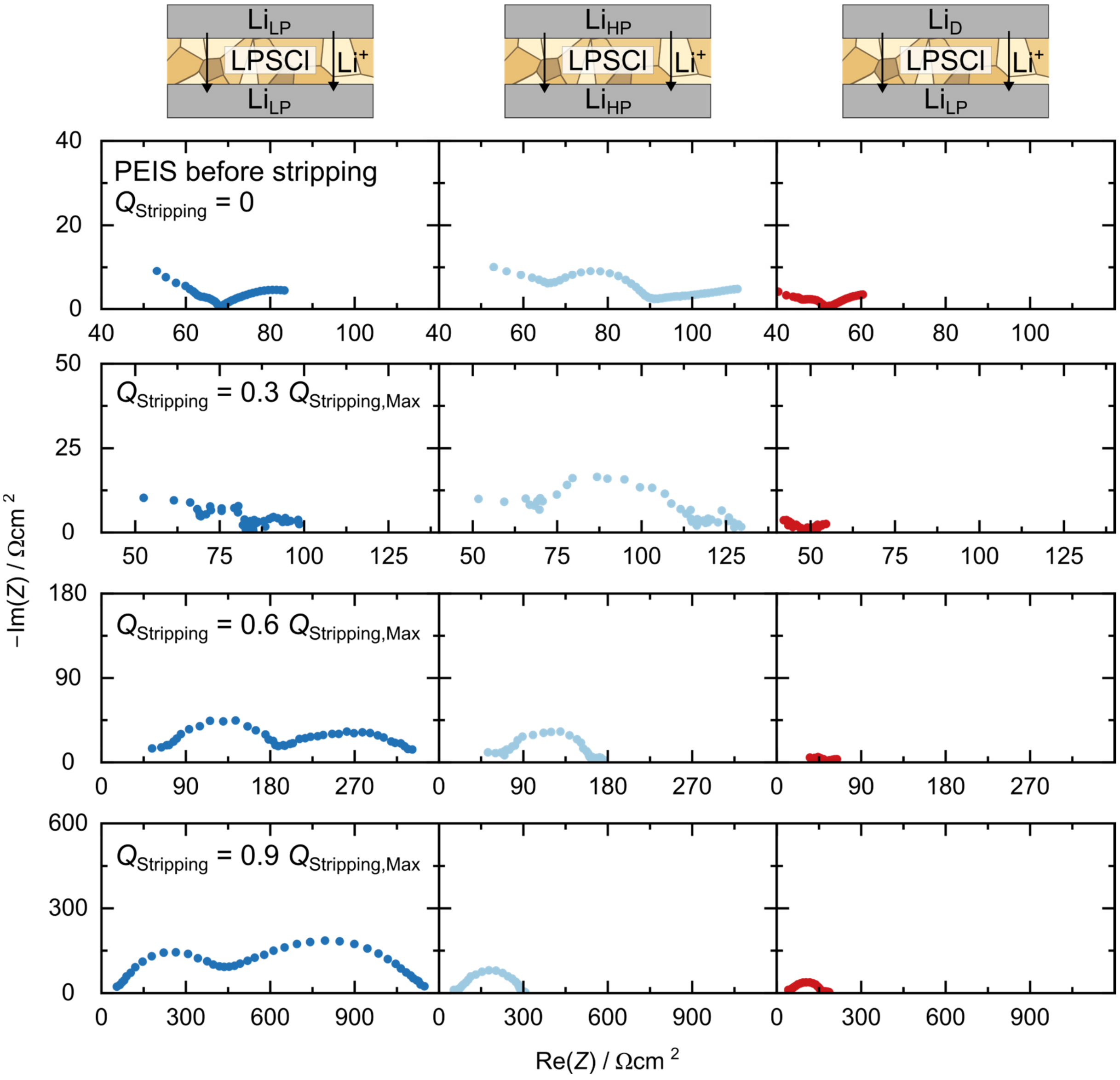

Figure S10: **Impedance analysis of symmetrical Li|LPSCl|Li cells.** Impedance spectra of three different Li|LPSCl|Li cells before and during lithium stripping in Nyquist representation. The cell setups with different lithium electrodes (Li_{LP}, Li_{HP}, and Li_D) are depicted above. As marked with the text, the first impedance spectra were recorded before applying a current using PEIS. The following spectra were recorded during the stripping process with an applied current of 100 µA cm^{-2} using GEIS. The times at which the spectra were recorded are given in fractions of the total stripping capacity $Q_{Stripping.Max}$. Please consider the different scalings of the graphs.

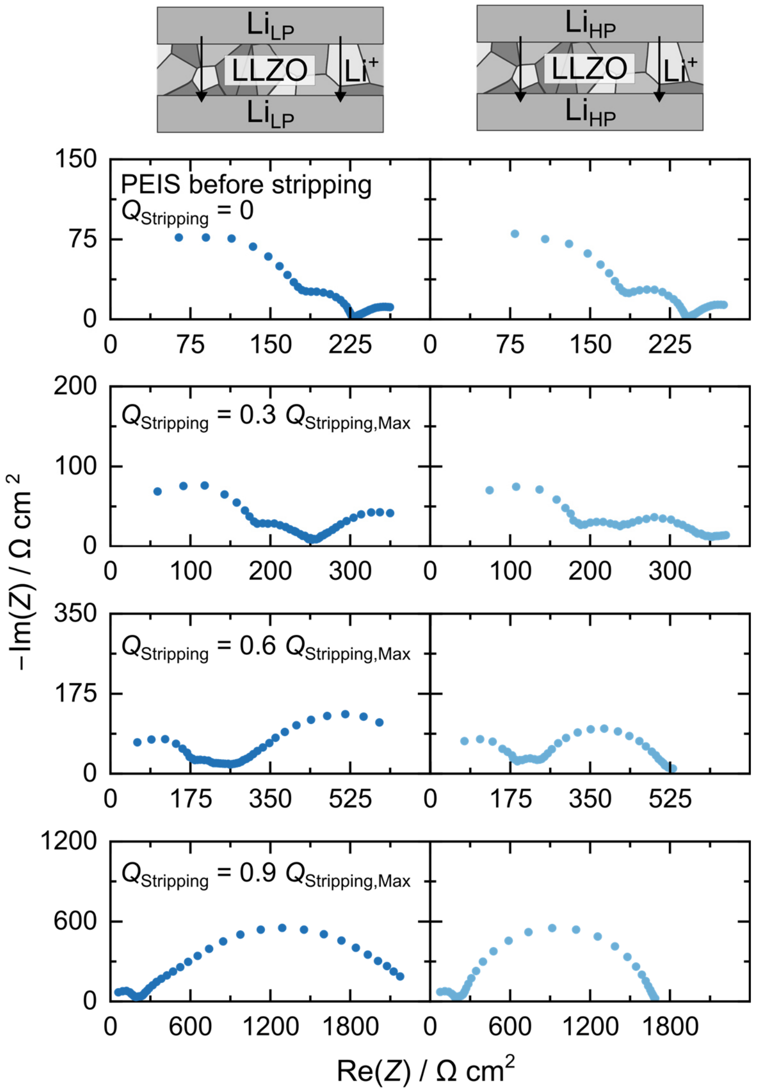

Figure S11: **Impedance analysis of symmetrical Li|LLZO|Li cells.** Impedance spectra of the symmetrical Li|LLZO|Li cells before and during lithium stripping in Nyquist representation. The cell setups with different lithium samples are depicted above. As marked with the text, the first impedance spectra were recorded before applying a current using PEIS. The following spectra were recorded during the stripping process with an applied current of 100 µA cm^{-2} using GEIS. The times at which the spectra were recorded are given in fractions of the total stripping capacity $Q_{Stripping.Max}$. Please consider the different scalings.

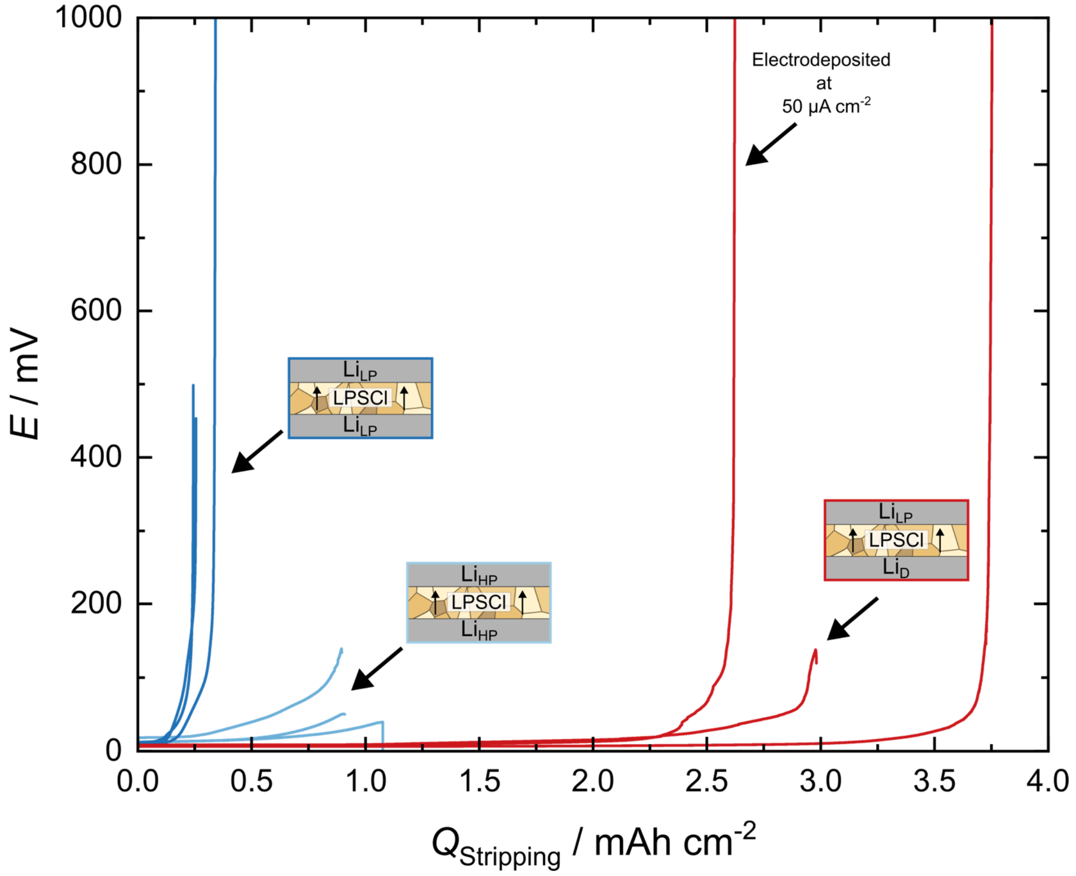

Figure S12: Reproduced cells using LPSCl as SE. Stripping curves of the cell systems examined using LPSCl as solid electrolyte. Three cells of type Li_{LP}|LPSCl|Li_{LP}, Li_{HP}|LPSCl|Li_{HP} and Li_{LP}|LPSCl|Li_{D} are shown. The respective cell system is shown schematically next to the corresponding stripping curves. In all cases a current density of 100 µA cm^{-2} was used for lithium stripping.

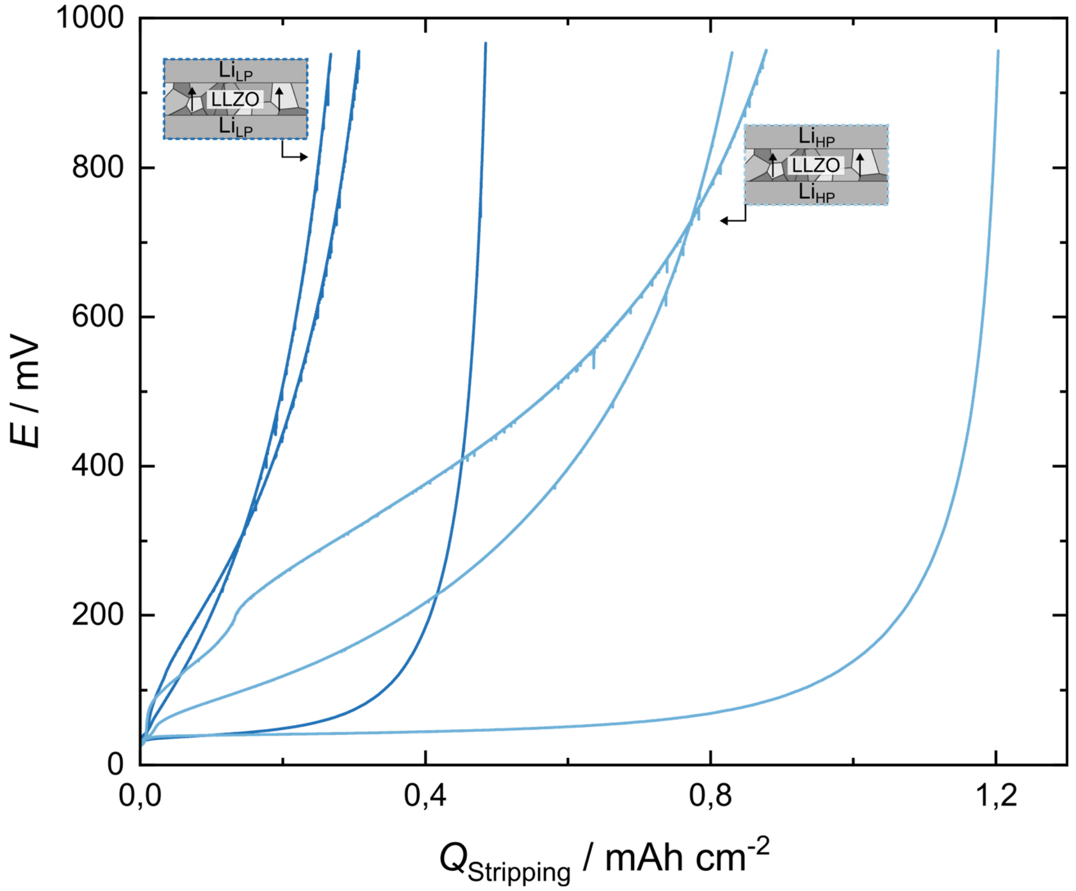

Figure S13: Reproduced cells using LLZO as SE. Stripping curves of the cell systems examined using LLZO as solid electrolyte. Three cells of type Li_{LP}|LLZO|Li_{LP} and Li_{HP}|LLZO|Li_{HP} are shown. The respective cell system is shown schematically next to the corresponding stripping curves. In all cases a current density of 100 µA cm^{-2} was used for lithium stripping.

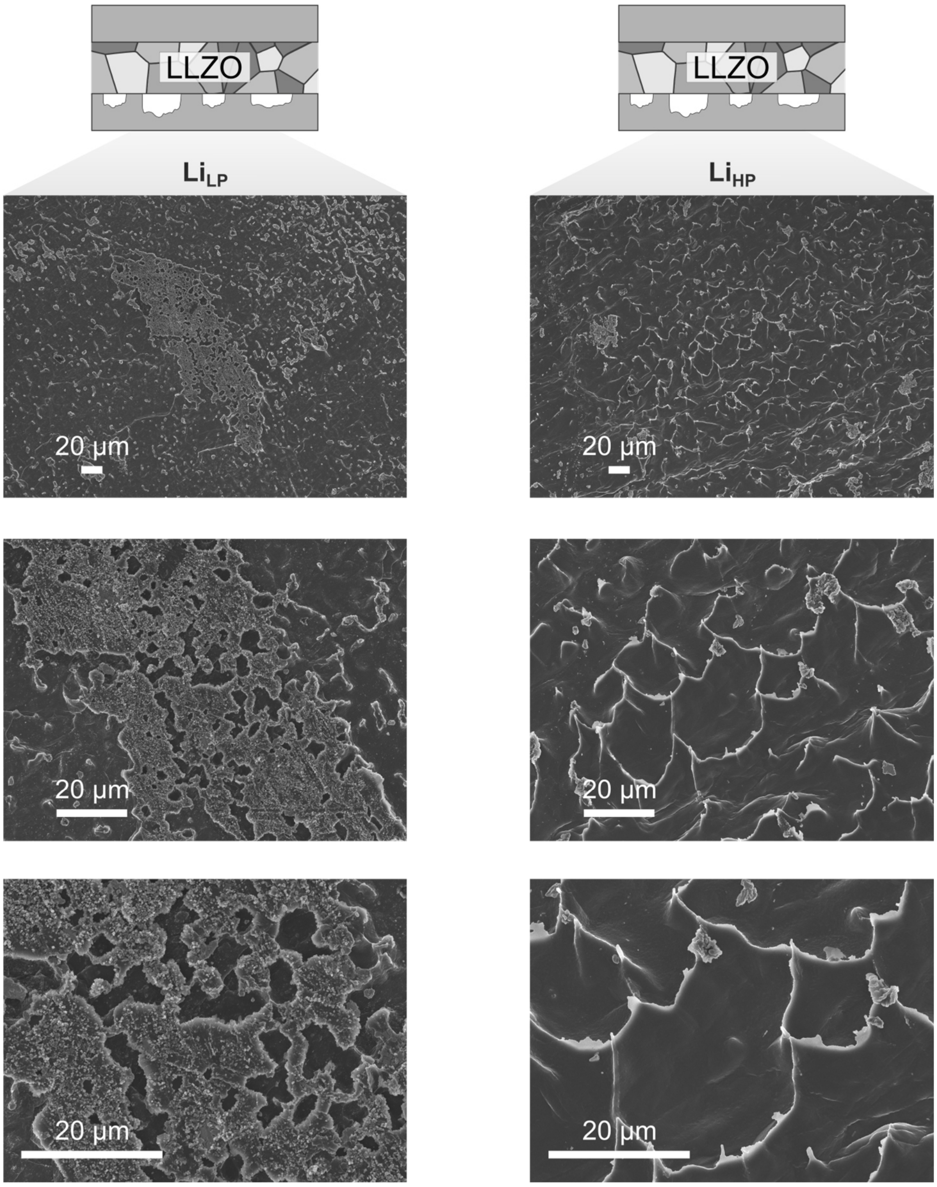

Figure S14: Pore morphology analysis of different lithium samples. Top-view SEM analysis of the stripped Li_{LP} and Li_{HP} electrodes from Figure 4 after contact loss. On the left side the Li_{LP} electrode can be seen, showing domains where seemingly remains of lithium are still present. On the right side the Li_{HP} electrode can be seen, showing almost no remaining lithium spots. The different panels show different magnifications. The sample was analyzed ex-situ after stripping Li_{LP} or Li_{HP} at a current density of 100 μA cm^{-2} for different capacities in symmetrical Li|LLZO|Li systems (see Figure 4).

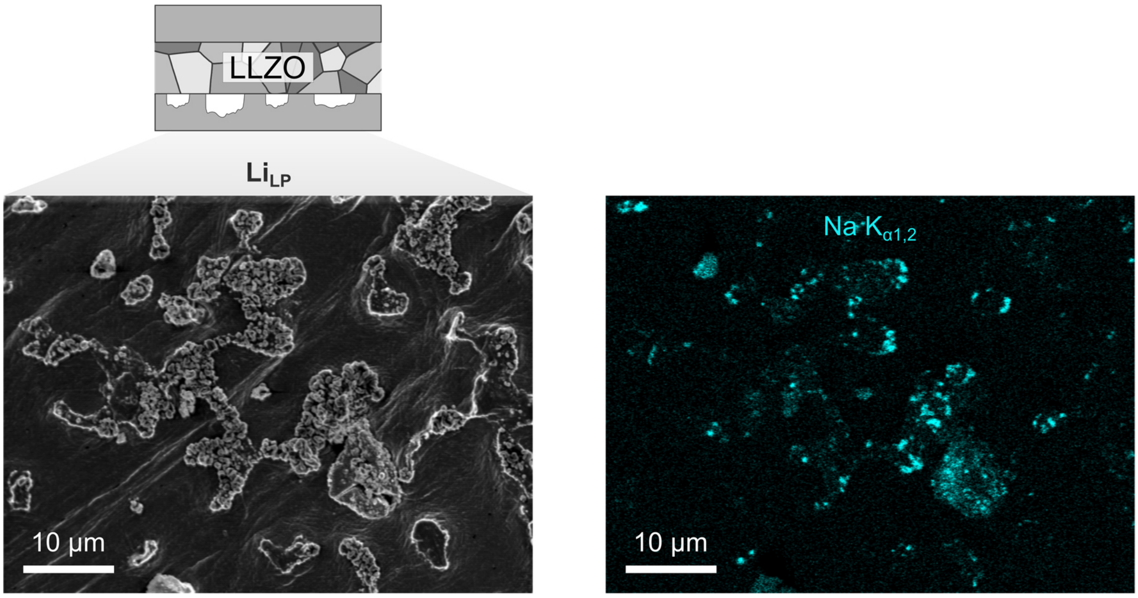

Figure S15: **EDX analysis of a stripped Li$_{LP}$ electrode.** Top-view SEM and EDX analysis of the stripped Li$_{LP}$ electrode from the Li$_{LP}$|LLZO|Li$_{LP}$ cell from Figure 4. On the left a SEM picture can be seen, showing some residual lithium after pore formation. The EDX map of the Na K$_{\alpha1,2}$ signal on the right shows an enrichment of sodium where lithium remains. The sample was analyzed ex-situ after stripping Li$_{LP}$ at a current density of 100 µA cm^{-2} for 0.48 mAh cm^{-2} in a symmetrical Li$_{LP}$|LLZO|Li$_{LP}$ systems (see Figure 4).

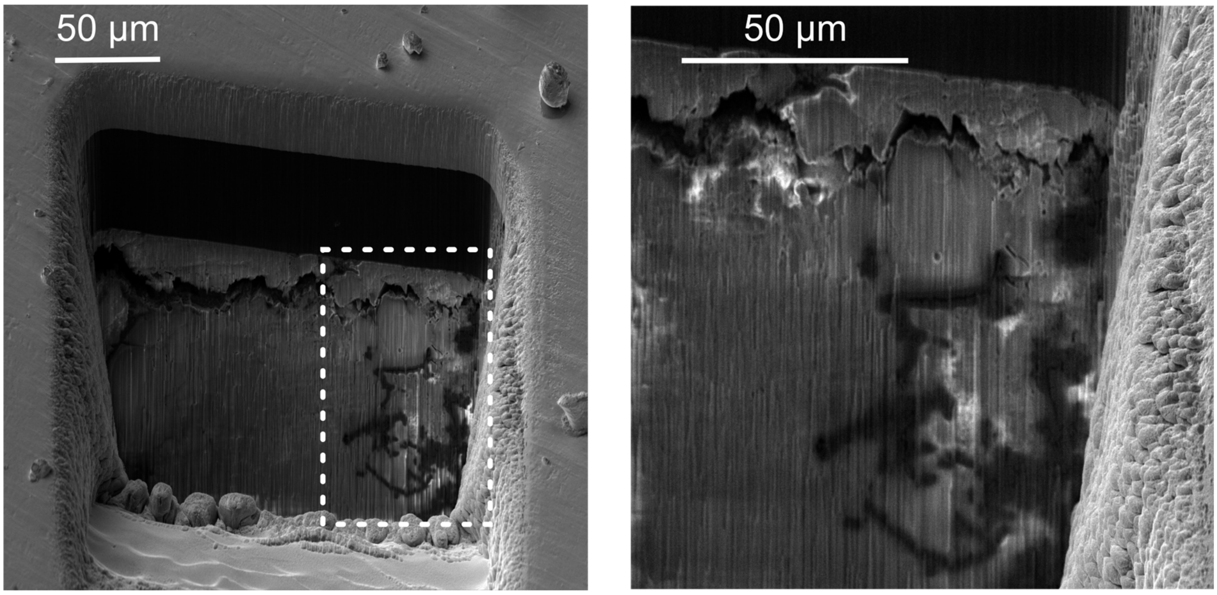

Figure S16: **Cross-section analysis of the steel|LPSCl interface after electrodeposition.** Cross-section of a Li$_{D}$|LPSCl interface similar to the sample from Figure 4. As highlighted with the dashed rectangle and with a higher magnification in the right image, dendritic structures are visible near the surface of the solid electrolyte. This sample was analyzed ex-situ after electrodeposition of 5 mAh cm^{-2} lithium at a current density of 100 µA cm^{-2} in a reservoir-free cell system on a steel electrode.

Supplementary Note 1

Sputtering effect on the resulting XPS spectra

The change in shape of the acquired spectra in Figure S2 and the change in calculated sodium concentration during sputtering with 10 kV indicates that lithium is preferentially sputtered, leading to an apparent increase in sodium. Moreover, Shard and Baker[1] have demonstrated that increasing the accelerating voltage while maintaining a constant argon cluster size can enhance the removal of inorganic degradation products during argon cluster sputtering, which could explain the different shape of the XPS spectrum after the final sputtering step with an acceleration voltage of 20 kV.

Supplementary Note 2

Binary phase diagram of the Li-Na system

As can be seen from the phase diagram in Figure S6, only a very low amount of sodium can be dissolved homogeneously in solid lithium at room temperature. Accordingly, lithium production from a melt, inevitably containing sodium as an impurity, leads to sodium precipitation. The amount, size and distribution of the sodium precipitates will depend on the specific sodium content and processing route.

Supplementary Note 3

Residue analysis on the LLZO surface after Li_{LP} stripping using XPS and ToF-SIMS.

Using ToF-SIMS, a significant increase of the signal intensity for Na^+ on the grey residues compared to the pristine surface can be observed in Figure S7.. In line with this, a strong enrichment of sodium is observed in the former contact area of the lithium electrode using XPS, which supports the SIMS results. From this we conclude that during stripping there is an accumulation of sodium at the interface to the LLZO pellet, which appears in the form of the gray residues.

Supplementary Note 4

Reproduced cells using LPSCl and LLZO as SEs.

Despite minor deviations within the respective cell systems, it is clear that the stripping capacities of $Li_D > Li_{HP} > Li_{LP}$. The drawn conclusions of the manuscript are therefore supported. At this point we like to mention that the stripping curve marked "Electrodeposited at 50 µA cm^{-2}" was electrodeposited at a current density of 50 µA cm^{-2}. The other two Li_D samples were electrodeposited at a current density of 100 µA cm^{-2}. This observed trend is also confirmed by the measurements using LLZO as SE. Here too, there is an increase in the lithium stripping capacity of Li_{HP} compared to Li_{LP}. All layers were dissolved at a current density of 100 µA cm^{-2}.

Supplementary References

(1) Shard, A. G.; Baker, M. A. Practical Guides for X-Ray Photoelectron Spectroscopy: Use of Argon Ion Beams for Sputter Depth Profiling and Cleaning. *Journal of Vacuum Science & Technology A* **2024**, *42* (5). https://doi.org/10.1116/6.0003681.

(2) Shard, A. G. Detection Limits in XPS for More than 6000 Binary Systems Using Al and Mg Kα X-rays. *Surface and Interface Analysis* **2014**, *46* (3), 175–185. https://doi.org/10.1002/sia.5406.

(3) Zhang, S.; Shin, D.; Liu, Z. K. Thermodynamic Modeling of the Ca-Li-Na System. *CALPHAD* **2003**, *27* (2), 235–241. https://doi.org/10.1016/j.calphad.2003.09.001.
